# Dermatology Program Coordinators’ Role and Insight in the Resident Selection Process

**DOI:** 10.7759/cureus.71640

**Published:** 2024-10-16

**Authors:** Patricia K Mansfield, Rylee Moody, Joey Weissmann, Matthew Simpson, Nicole Burkemper, Gillian Heinecke

**Affiliations:** 1 Department of Dermatology, Saint Louis University School of Medicine, St. Louis, USA; 2 Department of Dermatology, Saint Louis University, SLUCare SSM Health, St. Louis, USA; 3 Advanced HEAlth Data (AHEAD) Institute, Saint Louis University, St. Louis, USA

**Keywords:** interview, program coordinators, program directors, residency application, resident selection, resident selection committee, survey

## Abstract

Background: With the evolution of residency applications and the myriad of roles that program coordinators (PCs) undertake, there is a gap in the medical education literature surrounding PCs’ role in the resident selection process.

Objective: We sought to quantify the role of Dermatology residency PCs in the resident selection process and the unique perspective they gain from their interactions with applicants.

Methods: PC- and program director (PD)-specific surveys were designed and distributed in 2023 via email to Dermatology PDs via the Association of Professors of Dermatology (APD) listserv and to PCs via email using the contact information found on publicly available residency program websites.

Results: Survey responses included 62 PCs (33.9% response rate; sent to 184 PCs) and 35 PDs (unable to ascertain response rate; sent to APD listserv). Of the PC respondents, 25 (40.3%) reported participation in applicant ranking, with six (24%) participating as formal committee members, 14 (56%) contributing informal commentary, and only five (20%) functioning in administrative-only roles. Further, 16 (64%) PCs who participated in applicant ranking reported that their input yielded a change in the candidate ranking order, and a majority agreed that their input was helpful (21, 84%) and valued (21, 84%). All eight (100%) PDs who included their PC in the applicant ranking agreed that the PC input was helpful and valued. Finally, 49 (79.1%) PCs agree that they gain a perspective on candidates, with 36 (58%) feeling that their perspectives are unique from those of faculty members.

Conclusion: Residency PCs have the potential to play a reportedly consequential, helpful, valuable, and unique role in the resident selection process.

## Introduction

The Accreditation Council for Graduate Medical Education (ACGME) oversees accreditation for graduate medical training programs for resident physicians in the United States. The ACGME program requirements outline the roles of residency program directors (PDs), physicians who hold authority and accountability for the operations of the residency program, and program coordinators (PCs), individuals with inconsistent formal training backgrounds who provide leadership, resident/personnel management, and administrative assistance within the residency program. While all programs must have a residency PC [1], the roles and responsibilities of PCs vary across different residency training programs. A prior survey study evaluated the training and general responsibilities of PCs in neurosurgery residency programs across the United States, highlighting the variety of formal/informal training and education these individuals receive and their various administrative duties in department management and accreditation [2]. In this study, 97% of PCs listed interview scheduling as one of their current duties [2]. No prior study, however, has specifically evaluated the degree to which PCs are involved in the resident selection and interview process. In our experience, the PCs' role in assisting in this resident selection process is common in Dermatology.

In Dermatology, the volume of residency program applications has recently increased nationally [3]. The resident application process itself is evolving, including changes to the application format and the introduction of program signaling [4]. With the complexity of the resident selection process and the myriad of roles and responsibilities that PCs undertake, we sought to address a gap in the medical education literature surrounding PCs’ roles in the resident selection process.

The objective of our survey study was to investigate and quantify the specific role of Dermatology residency PCs in the resident selection process and the unique perspective they gain from their interactions with applicants. This survey study expands upon existing literature documenting the roles and responsibilities of PCs in academic departments [5], more specifically identifying the role of PCs in the resident application and selection process. As the first study in medical education literature to outline the current role of PCs in resident application and selection, we offer insight into the potential for increased utilization of these individuals in resident selection.

## Materials and methods

Utilizing input from SSM Health Saint Louis University School of Medicine PDs and PC, research team members designed a 27-question PC survey (Table 1) highlighting the PCs’ own experiences and a 22-question PD (Table 2) survey highlighting PDs’ perception of PC involvement in their residency programs. These surveys were distributed in 2023 via e-mail to Dermatology residency PDs using contact information found on the Association of Professors of Dermatology (APD) listserv and to PCs via email using contact information found on publicly available residency program websites. Data analysis included calculating counts and percentages of responses for specific survey questions among the two respondent groups (PCs and PDs).

**Table 1:** Program coordinator survey questions GED: General educational development

Program coordinator question	Answer options
Have you worked as a program coordinator during at least 1 residency interview season?	Yes
	No
Where is your program located?	Pacific West: AK, CA, HI, OR, WA
	Mountain West: AZ, CO, ID, MT, NM, NV, UT, WY
	West North Central: IA, KS, MN, MO, ND, NE, SD
	East North Central: IL, IN, MI, OH, WI
	West South Central: AR, LA, OK, TX
	East South Central: AL, KY, MS, TN
	South Atlantic: DC, DE, FL, GA, MD, NC, SC, VA, WV
	Middle Atlantic: NJ, NY, PA
	New England: CT, MA, ME, NH, RI, VT
How many years have you been a program coordinator?	Less than 1 year
	1-2 years
	3-4 years
	5-9 years
	10-14 years
	15 years or longer
How many program coordinators does your Dermatology program have?	1
	2
	3
	4 or more
How many residents in total are in your program?	1-6
	7-12
	13-18
	19 or more
Do you oversee only the Dermatology residency program or do you oversee multiple residency programs (Internal Medicine, Pathology, etc.)?	1 (only Dermatology)
	2 programs
	3 or more programs
Which parts of the Dermatology program do you oversee?	Only the core residency program
	Only fellowship (Dermatopathology, Mohs, etc.)
	Both core residency and fellowship
What is your highest level of education?	Less than high school
	High school/GED
	Some college
	2-year degree (Associate’s)
	4-year degree (BA/BS)
	Master’s
	Doctoral
How many residency applications does your program receive each year?	Less than 200
	200-299
	300-399
	400-499
	500-599
	600 or more
How many applicants does your Dermatology residency program interview?	Less than 20
	20-29
	30-39
	40-49
	50-59
	60 or more
Who is listed as the program contact on your website?	Program coordinator
	Program director
	Both program director and coordinator
	Generic program email
	No contact given
	Other
Do you have a role in the resident selection process?	Yes
	No
Do you review applications for applicant selection to interview?	Yes
	No
	Prescreen applicants with filters but do not review content of the application
Do you interview applicants?	Yes
	No
Do you participate in any way in the applicant ranking?	Yes
	No
How do you participate in the applicant ranking?	As a formal member of the committee
	Contributing informal commentary
	Only in an administrative assistant role
Do you feel like your input on applicant ranking is helpful?	Strongly agree
	Agree
	Neutral
	Disagree
	Strongly disagree
Do you feel like your input on applicant ranking is valued?	Strongly agree
	Agree
	Neutral
	Disagree
	Strongly disagree
Has your input changed the ranking order of candidates?	Yes
	No
What is the maximum ranking order change your input resulted in?	1-5
	More than 5
How has your input changed the ranking order of candidates?	My input has moved an applicant’s ranking UP
	My input has moved an applicant’s ranking DOWN
	My input has moved applicants’ rankings both UP AND DOWN
How has your input significant changed the ranking order of candidates?	My input has moved an applicant’s ranking UP more than 5 positions
	My input has moved an applicant’s ranking DOWN more than 5 positions
	My input has moved applicants’ ranking BOTH up and down more than 5 positions
Was an applicant not ranked because of your input?	Yes
	No
Do you feel you gain a perspective on the candidates from the interactions you have with them?	Strongly agree
	Agree
	Neutral
	Disagree
	Strongly disagree
What factors influence your perception of an interview candidate? (Check all that apply)	Email correspondence
	Phone correspondence
	Face-to-face or in-person interactions
	Timeliness on interview day
	Other
Please specify the other factor(s) that give you a unique perspective not listed above	(Free response)
Do you think virtual interviews have positively/negatively influence your perception of candidates compared to in-person interviews?	Positively
	Negatively
	Both positively and negatively
Comments	(Free response)

**Table 2:** Program director survey questions

Program director question	Answer options
Have you worked as a program director during at least 1 residency interview season?	Yes
	No
Where is your program located?	Pacific West: AK, CA, HI, OR, WA
	Mountain West: AZ, CO, ID, MT, NM, NV, UT, WY
	West North Central: IA, KS, MN, MO, ND, NE, SD
	East North Central: IL, IN, MI, OH, WI
	West South Central: AR, LA, OK, TX
	East South Central: AL, KY, MS, TN
	South Atlantic: DC, DE, FL, GA, MD, NC, SC, VA, WV
	Middle Atlantic: NJ, NY, PA
	New England: CT, MA, ME, NH, RI, VT
How many years have you been a program director?	Less than 1 year
	1-2 years
	3-4 years
	5-9 years
	10-14 years
	15 years or longer
How many program directors does your Dermatology program have?	1
	2
	3
	4 or more
How many residents in total are in your program?	1-6
	7-12
	13-18
	19 or more
How many residency applications does your program receive each year?	Less than 200
	200-299
	300-399
	400-499
	500-599
	600 or more
How many applicants does your Dermatology residency program interview?	Less than 20
	20-29
	30-39
	40-49
	50-59
	60 or more
Who is listed as the program contact on your website?	Program coordinator
	Program director
	Both program director and coordinator
	Generic program email
	No contact given
	Other
Do you have a role in the resident selection process?	Yes
	No
Do you review applications for applicant selection to interview?	Yes
	No
	Prescreen applicants with filters but do not review content of the application
Do you interview applicants?	Yes
	No
Does your program coordinator have a role in the resident selection process?	Yes
	No
Does your program coordinator participate in any way in the applicant ranking?	Yes
	No
How does your program coordinator participate in the applicant ranking?	As a formal member of the committee
	Contributing informal commentary
	Only in an administrative assistant role
Do you feel like your program coordinator’s input on applicant ranking is helpful?	Strongly agree
	Agree
	Neutral
	Disagree
	Strongly disagree
Do you feel like your program coordinator’s input on applicant ranking is valued?	Strongly agree
	Agree
	Neutral
	Disagree
	Strongly disagree
Do you feel your program coordinator has a unique perspective on the candidates that the other faculty members do not experience?	Strongly agree
	Agree
	Neutral
	Disagree
	Strongly disagree
What factors do you think give your program coordinator a unique perspective on an interview candidate that other members of the residency selection committee do not experience? (Check all that apply)	Email correspondence
	Phone correspondence
	Face-to-face or in-person interactions
	Timeliness on interview day
	Other
Please specify the other factor(s) that give you a unique perspective not listed above	(Free response)
Do you think your perception of applicants has changed with virtual versus in-person interviews?	Yes
	No
	I have not experienced in-person interviews
	I have not experienced virtual interviews
Do you think virtual interviews have positively/negatively influenced your perception of candidates compared to in-person interviews?	Positively
	Negatively
	Both positive and negatively
Comments	(Free response)

## Results

Participant residency program demographics

Survey responses included 62 Dermatology PCs (33.9% response rate; sent to 184 PCs) and 35 PDs (unable to ascertain response rate; sent to APD listserv). Demographics for these 97 respondents and their affiliated programs (location, length of time in position, size of program) are outlined in Table 1. Most survey respondents (94.3% of PCs and 91.9% of PDs) function in Dermatology residency programs with only one PC. The residency programs of PC and PD respondents varied in size. A majority of PC respondents (66.1%) oversee only the Dermatology residency program, as opposed to overseeing multiple residency programs (i.e., Internal Medicine, Pathology, etc.). Further, 50% of PC respondents oversee only the core residency program, while 48.4% oversee both the core residency and fellowship and 1.6% oversee only a fellowship program (Dermatopathology, Mohs, etc.). Specifically highlighting the reported education levels of Dermatology residency PCs, a majority of PC participants (59.7%) have received a four-year degree or higher (32.3% four-year degree; 24.2% Master’s; 3.2% Doctoral). Further, 37.1% of PC participants received less than a four-year-degree (16.1% two-year degree; 14.5% some college; 6.5% high school/GED) (Table 1).

Current role of PCs in resident selection and ranking

Initial survey questions sought to identify the general role of PCs in the resident selection process. Of the 62 PC respondents, 48.4% reported that they play a role in the resident selection process. A total of 56.5% prescreen applicants with filters without reviewing the content of the application, and 14.5% review the content of applications for selection to interview. A small number (4.8%) interview the applicants themselves. Of the 35 PD respondents, 40% reported that their PCs play a role in the resident selection process (Table 2).

To further quantify the involvement of PCs in selecting residents for their program, we tailored many of our questions toward the applicant ranking portion of the residency application process. Of the 62 PC respondents, 40.3% (n = 25) reported that they participated in the applicant ranking. Further, of these 25 PCs who reported that they do participate in ranking, 24% participate as formal members of the selection committee, 56% contribute informal commentary, and 20% serve only in an administrative role. A majority (64%) of these 25 PCs reported that their input has changed the ranking order of candidates. More specifically, 62.5% of those who altered the ranking order moved an applicant 1-5 spots up or down on the final rank list, and 37.5% moved an applicant >5 spots on the final rank list. Of the 35 total PD respondents, only 22.9% (n = 8) reported that their PCs participate in applicant ranking, with 100% of these PDs reporting that their PCs participate by contributing informal commentary (as opposed to functioning as formal selection committee members or solely as administrative assistants) (Table 2).

Quality and perspective of PC input in resident selection

In an attempt to measure the quality of PC input in the resident selection process, we asked PC and PD respondents to assign Likert scale qualifiers (strongly agree, agree, neutral, disagree, strongly disagree) to the helpfulness, value, and uniqueness of PC input. Of the 25 PC respondents who reported that they participate in applicant ranking, 84% agreed or strongly agreed that their input is helpful and valued by their program. Of the eight PD respondents who reported that their PCs participate in applicant ranking, 100% agreed or strongly agreed both that their PC’s input is helpful and valued (Table 2).

To provide meaningful input that is distinct from that of faculty members in the resident selection process, PCs would presumably need to (1) gain a perspective on candidates (based on the degree of their interactions) and (2) gain a unique perspective on these candidates that faculty members may not encounter in the interview process. A majority (79.0%) of PC respondents agreed or strongly agreed that they gain a perspective on candidates based on the interactions they have with these individuals, and their perspectives are influenced by email correspondence (89.8%), phone correspondence (61.2%), in-person interactions (69.4%), and timeliness on interview day (61.2%). More specifically, 58.1% of PC respondents agreed or strongly agreed that they gain a unique perspective on candidates that faculty members do not experience. Factors that provide these PCs with unique perspectives that faculty members do not experience included, similarly, email correspondence (97.2%), phone correspondence (75%), in-person interactions (69.4%), and timeliness on interview day (58.3%). Of note, PD responses regarding the perspectives of their PCs on candidates were similar. A majority (71.4%) of PD respondents agreed or strongly agreed that their PCs gain a perspective on candidates, and 68.6% agreed or strongly agreed that their PCs gain a unique perspective on candidates that faculty members do not experience, citing similar factors/interactions that contribute to these PC perspectives (email/phone correspondence, in-person interactions, timeliness on interview days) (Table 3).

A note on virtual interviews and perception of applicants

Surveys were distributed to PDs and PCs after the 2022-2023 application cycle, during which the APD, in alignment with the Association of American Medical Colleagues (AAMC), recommended the utilization of virtual formats for interviews and recruiting activity to promote equity in the application process [5]. In our study, PC and PD respondents had varied opinions regarding virtual interviews and the impact that this format has on their perception of applicants. Among the 50 PCs (80.6%) who indicated they had experienced both in-person and virtual interviews, 54% (n = 27) selected that their perception of applicants has been altered by virtual interviewing. For the 27 PCs that indicated a change, most responded that the virtual format skewed their perceptions of applicants both positively and negatively (55.6%), followed by negatively (37%) and positively alone (7.4%). All of the 35 PD respondents had experienced both in-person and virtual interviews, and 54.3% (n = 19) reported that their perception of applicants has been changed with the onset of virtual interviewing. For the 19 PDs that indicated a change, most responded that the virtual format skewed their perceptions of applicants both positively and negatively (68.4%), while the rest responded that their perceptions had been skewed negatively alone (31.6%). Of note, four out of five PD free responses and 11 out of 20 PC free responses demonstrated negative attitudes toward virtual interviewing in the resident selection process.

## Discussion

From these results, we can identify that many PCs in Dermatology residency programs across the United States play a role in the resident application and selection process. PC perspective and input in the resident selection are reportedly consequential, as their informal and formal input can alter the final match list ordering. PC input and the PC perspective surrounding residency applicants are reportedly unique from the input and perspectives of faculty members, due to factors including phone/email correspondence, in-person interactions, and timeliness on interview day. Further, PC input is identified as helpful and valued by PDs and PCs alike. However, there is a discrepancy between PC perception of how valued their input is (84% of PCs agreeing that their input is valued) in comparison to the 100% of PD respondents who reported that they value PC input. This discrepancy could point to a lack of positive reinforcement by PDs regarding the value that they place on their PCs’ input or to a lack of PC awareness regarding how their input is utilized specifically in the resident selection process.

To our knowledge, this study is the first to outline the specific roles of ACGME residency PCs in the resident selection process in any general or subspecialty field. Previously published studies in the medical literature have outlined general administrative roles and educational backgrounds of residency PCs in orthopedic surgery [6] and neurosurgery [2] programs. Specifically, 69% of orthopedic surgery residency PCs reported that they had total responsibility for or coordinated the application process [6], and 98.7% of neurosurgery residency PCs reported that they scheduled interviews [2], highlighting more general roles of these individuals in the applicant selection process. Notably, our study delineates these roles by identifying more specific ways in which PCs interact with applicants and play a role in their selection for training at their residency program.

This study is limited by an uneven distribution of survey respondents (62 PCs, 35 PDs), by the absence of linked PC-PD responses within specific programs to allow for within-program correlation (excluded in protocol to preserve participant anonymity), and by a pool of participants restricted to respondents in dermatology residencies. Further investigation of these trends in residency programs within other fields is warranted.

## Conclusions

This study quantifies the current roles of PCs in resident selection in Dermatology programs and identifies the potential for PCs to play an important role in the resident selection process. Regarding this new and previously unreported data, residency PDs and faculty members should be aware of the current PC- and PD-reported roles of PCs in the selection of residents for Dermatology residency programs across the United States. These leaders in residency programs can also note the potential for PCs to play a reportedly consequential, helpful, valuable, and unique role in the Dermatology resident selection process. This project serves as a springboard for future research surrounding the potential roles and impact of PCs in resident selection in other specialty and subspecialty fields.
